# Individualized Remotely Supervised Motor Activity Programs Promote Rehabilitation Goal Achievement, Motor Functioning, and Physical Activity of People with Rett Syndrome—A Single-Cohort Study

**DOI:** 10.3390/ijerph20010659

**Published:** 2022-12-30

**Authors:** Alberto Romano, Elena Ippolito, Martina Favetta, Meir Lotan, Daniel Sender Moran

**Affiliations:** 1 Department of Health System Management, Ariel University, Ariel 4070000, Israel; 2 Airett Innovation and Research Center, 37122 Verona, Italy; 3 SMART Learning Center, 20133 Milan, Italy; 4 Movement Analysis and Robotics Laboratory, Intensive Neurorehabilitation and Robotics Department, Bambino Gesù Children’s Hospital, 00165 Rome, Italy; 5 Department of Physiotherapy, Ariel University, Ariel 4070000, Israel; 6 Israeli Rett Syndrome National Evaluation Team, Ramat Gan 5200100, Israel

**Keywords:** Rett syndrome, telerehabilitation, home exercise program, motor skills, physical activity, physical therapy modalities

## Abstract

Background: Gross motor function in Rett syndrome (RTT) is always limited. The complex clinical picture typical of most people with RTT requires intensive and specific rehabilitation programs. Previous reports on remotely supervised motor activity programs suggested positive outcomes for this population. The current article describes the impact of a remote-supervised motor activity program carried out by family members of individuals with RTT on achieving rehabilitation goals and improving gross and fine motor functioning and daily physical activity. Methods: Forty subjects with RTT followed a three-month remotely supervised motor activity program carried out by their family members at home after a three-month baseline period. After the end of the intervention, a three-month wash-out period was implemented. Rehabilitation goal achievement, motor functioning, and level of daily physical activity were measured. Results: 82.4% of rehabilitation goals were achieved or overachieved. Participants’ motor functioning and physical activity significantly increased after the intervention (*p* ˂ 0.001). Improvements were maintained after the wash-out phase. Conclusions: The proposed intervention was effective for people with RTT of various ages and severity levels. The results highlight the need for lifelong, individualized, daily based, and professionally supervised rehabilitation possibilities for individuals with RTT.

## 1. Introduction

Rett syndrome (RTT) is a severe neurodevelopmental disease affecting about 1/10,000 females, although a higher incidence rate has been reported by some researchers [1,2]. RTT is characterized by a regression of abilities in intellectual functioning, fine and gross motor skills, and communicative ability, which occurs after an apparently typical prenatal and perinatal period. Other features include the development of stereotypical hand movements, seizures, disturbed breathing patterns, scoliosis, growth retardation, ataxia, apraxia, and gait disturbances [3]. Gross motor function in RTT is always limited [4]. In the regression phase, patients begin to develop difficulties in coordination and balance that co-occur with uncontrolled movements of body segments and trunk [5,6,7]. Residual gross motor functions are usually preserved in older ages. Most adult patients maintain the ability to walk with support, and almost half can walk independently or with minimal support [8]. However, a further decrease in motor functioning quality with a progressive increase in the support needed is observed from the age of 13 [9,10]. Moreover, fluctuation in muscle tone has been reported from early childhood [11]. Neuromuscular impairment and musculoskeletal abnormalities have been reported in RTT syndrome. Such deformities of the spine and feet are prevalent, although all body joints can be affected [12]. 

Within such health-related complexities, the affected patients rely on early and appropriate diagnosis, adequate care at specialized centers, and multidisciplinary clinical follow-up [13]. Moreover, the complex clinical picture typically presented by most people with RTT requires intensive and specific rehabilitation intervention programs [14]. Primary caregivers, such as care providers at educational and rehabilitation centers, and parents and family members, play a vital role in supporting the health and wellbeing of children with disabilities, including those with RTT. Family involvement in child management increases the therapeutic intervention’s consistency [15]. 

Furthermore, as physical interventions for the person with RTT mainly aim to compensate for and reduce physical impairments, the therapeutic intervention should go beyond what is administered in the rehabilitation room or during individually applied sessions. Home intervention programs have shown positive effects in supporting functionality and reducing the mortality of people with Alzheimer’s disease and related disorders while reducing the stress experienced by caregivers [16]. In a randomized controlled trial, a home intervention program aimed at skill generalization and family member training was proposed for children with autism, global developmental delay, and language delay. The results showed more remarkable cognitive development and behavior changes for those children who received the additional intervention [17]. A Cochrane review on the effectiveness of home-based versus center-based physical activity programs on the health of older adults with cardiovascular risk factors, existing cardiovascular disease, chronic obstructive airway diseases, or osteoarthritis was conducted. The authors reported that home programs provide better results in terms of adherence to the prescribed exercises, especially in the long run [18]. The literature was recently enriched with studies proposing intensive home-based interventions for people with RTT conducted by family members and remotely supervised. Romano et al., 2021 described the effects of a three-month home-based, family-centered, individualized motor rehabilitation program carried out by family members and remotely supervised by expert therapists on the motor functioning and musculoskeletal abnormalities of a group of girls and women with RTT [13]. The authors reported the achievement of 78.7% of the stated rehabilitation goals, improvement in the participants’ motor functioning, and a high level of caregivers’ satisfaction with the intervention. A coherent high level of family members’ satisfaction with a similar intervention was reported by Lotan and colleagues (2021), along with motor functioning improvement [19]. Zwilling et al., 2022 suggested that a remote supervised activity program supported the wellbeing of parents of people with RTT during the Italian COVID-19 lockdown [20]. Moreover, an intense remotely supervised postural and activity program limited the scoliosis progression in a group of patients with RTT, leading in some cases to improvement in the spine asymmetry [21]. Furthermore, Fabio et al., 2022 evaluated the impact of two remote rehabilitation systems, concluding that an advanced telerehabilitation platform could provide better results than basic telerehabilitation [22]. Moreover, Dovigo and colleagues (2021) reported the positive results obtained with a remotely provided school service for the cognitive and social skills of girls with RTT during the COVID-19 lockdown [23].

The current article aims to describe the impact of a remote-supervised motor activity program carried out by family members of girls and women with RTT on achieving preset rehabilitation goals, gross and fine motor functioning, and improving the daily level of physical activity.

## 2. Materials and Methods

### 2.1. Ethical Issues

The Ariel University’s Institutional Review Board approved the study (no. AU-HEA-ML-20190326-1) that was conducted according to the ethical principles of the Helsinki Declaration and local regulations. All details relating to the study procedure were discussed with the participants’ parents or legal representatives, who signed an informed consent document. Enrolment was voluntary, with participants not receiving any incentives, financial or otherwise.

### 2.2. Experimental Design

A case series with multiple baselines A-A-B-A-A design was applied. Letters “A” represent the evaluation sessions conducted four times (T1, T2, T3, and T4), three months apart. Letter “B” represents the intervention phase conducted between T2 and T3 for three months. Multiple baselines were performed to allow comparison of the results achieved by participants during the intervention phase with those obtained within their pre-intervention therapeutic regimen and after three months from the suspension of the remote supervision. The intervention was family centered, and the study was conducted under the participatory action research (PAR) model. 

Family-centered care (FCC) can be described as placing the participants’ needs at the center of care, considering the context of their family and community. It requires the development of an individualized and dynamic model of care in strict collaboration with the child and family that will best meet these needs [24]. FCC is an approach to planning, delivering, and evaluating health care based on a mutually beneficial partnership among patients, families, and providers [25]. FCC puts the intervention emphasis on the child and family’s needs to support living patterns and be supported by the family’s strengths and resources [26]. The family is recognized as the constant in the participant’s life and the primary source of strength and support. Family members are involved in their child’s care and learn more about their child and treatment. In turn, they can share their knowledge with professionals, providing a more holistic picture of the child as part of the family [24]. Families are not referred to or treated as passive clients, receiving and implementing instructions suggested by others, as they play a fundamental role in the decision-making process concerning services for their child [27]. With the family unit as the primary and principal context for promoting the child’s health and wellbeing, FCC contributes to developmental progress and skill development [28,29]. 

PAR is a participatory, democratic process concerned with developing practical knowledge to pursue practical human purposes [30]. Action research involves clients (in this case, families and support staff of the person with RTT) throughout the research process and problem identification and disentangling. The PAR process involves numerous cycles of (a) assessment (of each participant’s therapeutic needs and family’s availability); (b) mutual goal attainment (intervention goals are constructed for each client together with the family regarding their feasibility and availability within the family’s framework); (c) action (the families and other support personnel implement the programs); (d) reflection (the program development is discussed over remote meetings by the supervisor and the parents); and (e) evaluation (the program achievements are discussed within the supervision meeting and modifications are applied if necessary). Action research assumes that the participants’ involvement empowers them with the final results of achieving more significant results. Action researchers seek change and develop solutions in collaboration with the client while maintaining sensitivity to the family’s needs and desires [31]. PAR design was selected as it is ideally suited to evolving programs within a highly personal collaborative framework, such as the one implemented in the current study. 

### 2.3. Participants

Participants in this study were girls and women with genetically confirmed classic RTT. Participants’ parents provide the medical report received at the time of the diagnosis of RTT containing the specific genetic mutation of the MECP2 gene. Participants and their families were recruited from the Italian Rett syndrome association (AIRett). To be included in this study, the participants’ parents had to have given their availability to carry out the activities foreseen in the therapeutic program for at least an hour a day for five days a week. Individuals affected by RTT variants and those who underwent surgery six months before recruitment were excluded from the present investigation. 

Forty-two participants and families complied with the inclusion criteria and were involved in the first evaluation (T1). Two families (4.8%) did not conclude the research protocol. One dropout was due to health problems of the participant’s mother that arose during the baseline period. The other dropout concerned a family with senior parents living in a rural area with negative involvement by local healthcare professionals giving the family contradicting advice regarding the suggested habilitation program. Therefore, the data analysis involved 40 participants. 

The RARS was used to evaluate the severity of RTT clinical manifestation at T1. The RARS is a 31-item RTT-specific severity scale [32]. Each item concerns a specific phenotypic RTT characteristic. The total score indicates the participant’s RTT severity level ranging from a mild deficit (reflected by lower scores) to severe symptoms (reflected by higher scores). A standardization procedure for the Italian population with RTT was conducted for the RARS. Skewness and kurtosis values, calculated for the total score distribution, were 0.110 and 0.352, respectively. The distribution was found to be normal. Internal consistency for the total score (0.912), as well as for the subscales, was high (0.811–0.934) [32,33].

Participants’ ages and RTT severity levels measured at T1 using the RARS are described in Table 1. The age, level of RTT severity, motor functioning level at baseline, and genetic mutation of each participant are provided as (Supplementary Material Table S1). 

At the initial evaluation, 11 participants were younger than 10 years, the age of 19 subjects was between 10 and 20 years, and 10 participants were older than 20 years. All participants resided at home with their parents. Seven participants followed a motor rehabilitative intervention for at least four hours a week, and 26 subjects attended such interventions between one and three hours per week. Five participants did not follow any motor rehabilitation treatment. All participants maintained the same rehabilitative intervention regimen for the duration of the current study.

### 2.4. Procedure

Before the intervention, informed consent was collected from all participants. Participants underwent four evaluation sessions at three-month intervals (±1 month, T1–T4). Each participant was assessed for passive joint mobility, gross motor skills, and average daily physical activity at each meeting.

**Figure 1:**
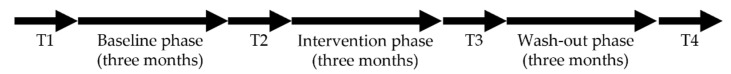
Procedure flowchart.

The study procedure flowchart is depicted in Figure 1. In the first evaluation meeting (T1), information related to participants’ clinical status (such as the presence of seizures, sleep disturbances, low bone density, and other conditions typically associated with RTT) and ongoing therapeutic interventions (such as physiotherapy, psychomotricity, hydrotherapy, and others) was collected. The individualized goals for the intervention phase were drafted and discussed with the participants’ parents and rehabilitation professionals during this meeting. The proposed goals referred to four areas: motor function, range of motion, hand functioning, and physical fitness. The intervention goals were identified following the SMART principle: Specific, Measurable, Attainable, Realistic, and Timely [34].

Between the first and second evaluation meetings, no changes were made to participants’ daily activities (baseline phase).

In the second evaluation session (T2), the rehabilitation goals were again discussed with each family and rehabilitation professionals and corrected, if necessary. Then, the Goal Attainment Scaling (GAS) was compiled for each identified goal. 

Between the second and third evaluation meeting (intervention phase), for each participant, an individualized program of simple therapeutic activities was drawn up and shared with the family. The programs aimed to achieve the identified therapeutic objectives through easily implemented physical activities performed within the girl’s daily life for about one non-continuous hour a day for five days a week. The participants’ families selected the weekly activity schedule according to their routines, availability, and habits. The activities included in the programs were related but were not limited to: (a) the maintenance of passive postures to prevent the onset or worsening of musculoskeletal abnormalities typically associated with RTT; (b) maintaining active symmetrical and asymmetrical postures to rebalance the trunk muscles and improve balance; (c) exercise of residual functional skills (e.g., sitting position, standing position, walking, postural passages, climbing/descending stairs); and (d) functional use of hands. The goals and activities identified were markedly different among participants in content, difficulty, and intensity to best suit each individual. Two weeks after the program delivery, which was needed for accommodation of the activities, a continuous cycle of fortnightly remote supervision was started and provided through a videoconference platform for one hour each until the end of the intervention phase. The first supervision meeting was mainly used to clarify doubts relating to the practical implementation of the activities proposed in the program. The subsequent supervision meetings were aimed at supporting the execution of the programs by answering the parents’ questions, adapting them to emerging needs, overcoming problems and obstacles, rearranging the timetable, adapting the proposed exercises, evaluating and sharing the achievement of the goals, and, if necessary, setting new goals. 

At the intervention end, during the third evaluation meeting (T3), the level of achievement of the treatment objectives established in the GAS was assessed.

The remote supervision meetings were suspended between the third and fourth evaluation sessions (wash-out phase). Within the wash-out phase, the families were encouraged to continue their program at their discretion. 

During the fourth evaluation meeting (T4), the GAS score for each rehabilitation goal was recollected.

### 2.5. Outcome Measures

#### 2.5.1. Goal Attainment Scaling (GAS)

The GAS was administered to assess the degree of achievement of the rehabilitation goals. This scaling system is a mathematical technique used in rehabilitation to quantify therapeutic goals’ achievement (or not) [35]. The GAS has been identified as the most sensitive tool to reflect and measure small changes in patients’ functioning and conditions that otherwise would not be found using standardized or physiological measures [36]. Rehabilitation goals were assigned with a weight ranging from 1 (mild) to 3 (substantial) to consider the relative importance of the goal to the participant and the difficulty that the rehabilitation team anticipates in achieving it [37]. The degree of achievement of each objective was assessed on a 5-point scale in a manner suitable for individuals with a potential to deteriorate as presented by those with RTT, a model suggested by King et al. (2000) [38]: a score of −1 represents the pre-intervention situation, and a score of −2 represents worsening compared to the pre-intervention assessment. If the patient reaches the goal at the expected level, the assigned value is 0. If obtained results exceed the experiments’ expectation, a +1 (a little more than expected) or +2 (much more than expected) value is given [38]. Each goal level was defined a priori by the experimenters and should be as objective and observable as possible. Each goal outcome score and weight were incorporated into a single aggregated T-score to obtain one numerical score for each patient regarding the rehabilitation goal achievement. The T-score was calculated by applying the following Formula (1) [35]:

(1) $$\mathrm{T-score}=50+\frac{10\cdot \sum \left(w_{i}x_{i}\right)}{\sqrt{\left[\left(1-\rho \right)\cdot \sum w_{i}^{2}+\rho \cdot {\left(\sum w_{i}\right)}^{2}\right]}}$$

where w_i_ = the weight assigned to each goal, x_i_ = the numerical value achieved for each goal (between −2 and +2), and ρ (rho) = the expected correlation of the goal scales. For practical purposes, ρ is commonly approximated to 0.3 [39]. Therefore, the composite goal score (sum of attainment levels × each goal’s relative weights) is transformed into a standardized measure (T-score) with a mean of 50 and a standard deviation of 10. A T-score represents an overall measure of the adequacy of the proposed goals. A T-score of 50 ± 10 indicates that the set goals were adequate to the intervention’s potential. A T-score > 60 suggests that the rehabilitation team has underestimated the treatment potential, and a T-score < 40 indicates that the objectives were overall overestimated for the intervention [35]. 

#### 2.5.2. Rett Syndrome Motor Evaluation Scale (RESMES)

RESMES was used to assess the gross motor functioning of all participants at each evaluation meeting. This tool is a 25-item RTT-specific scale investigating subjects’ gross motor performance across six sections: (i) standing, (ii) sitting, (iii) transitions, (iv) walking, (v) running, (vi) walking up/downstairs, for a total maximum score of 82 points. Higher scores correspond to lower motor functioning [40]. RESMES was validated on a sample of 60 females subjects with RTT (mean age: 12 years 5 months, SD: 8 years 9 months; range: 3–40 years) showing optimal inter-rater agreement among clinicians (s* statistic values always >0.70) and strong internal consistency (split-half reliability coefficient = 0.96 SD 0.18; Cronbach alpha coefficient for the entire scale = 0.95 CI 0.93–0.97) [33,41].

#### 2.5.3. Modified Bouchard Activity Record (mBAR)

This tool assessed the participants’ daily physical activity level. It is an RTT-specific whole-day diary card. Every 15 min of the day, a number can be assigned to the intensity of physical activity performed by the subject in the analyzed period. It comprises five activity levels adequate for the severe disability associated with RTT, ranging from “lying” to “walking at a vigorous intensity.” Higher scores correspond to higher physical activity levels [42]. A validation study of mBAR showed a strong correlation between the measured “uptime” (intended as time spent in standing and walking) with the number of steps taken during the day for the 43 females with RTT (mean age: 21 years, SD: 9 years) who can walk [42].

### 2.6. Statistical Analyses

As not all investigated variables were normally distributed, the non-parametric statistic was used. Wilcoxon signed-rank test was used to compare the GAS scores collected at T3 and T4 with the pre-intervention level (−1). Friedman’s test was run to compare the outcome measures scores obtained by participants at the four evaluation points. Post hoc analysis with Wilcoxon signed-rank test was conducted for pairwise comparisons. The effect size calculation for the above-reported repeated measures comparison (Friedman’s test) was performed with Kendall’s W coefficient. For the pairwise comparisons, the matched-pairs rank-biserial correlation was used [43,44]. An effect size between 0.140 and 0.310 was considered small, between 0.310 and 0.610 was judged medium, and above 0.610 was considered large [45]. A correlation analysis was performed to explore which aspects can influence the results of the proposed program. A relation between improvements in motor functioning and physical activity and the levels of therapeutic goals achievement (measured with GAS T-score) with participants’ age, severity level, motor functioning, and level of physical activity at baseline (T0) was investigated using the Spearman rank correlation coefficient. The threshold for significance for correlation analysis has been assumed as α = 0.05. No correction for multiple comparisons was applied [46].

## 3. Results

### 3.1. Goals Attainment

Among 40 subjects, 176 therapeutic goals were identified. A description of each goal is available within Supplementary Material Table S2. The amounts of goals identified for each area and the related GAS scores are described in Table 2. 

The average GAS value for all participants’ goals was +0.5 ± 1.0 at post-intervention evaluation (T3) and +0.4 ± 1.1 at follow-up evaluation (T4) with a statistical difference from the pre-intervention level (*p* < 0.001). On the other hand, no statistical difference was found between GAS scores collected at T3 and T4 (*p* = 0.337). At T3, 145 (82.4%) goals were achieved or overachieved (GAS score above 0), and at T4, 141 (80.1%) goals were scored at that level or above. Among 176 goals, the GAS score of 35 (19.9%) goals improved between T3 and T4. At the same time, 43 (24.4%) GAS scores were found to reduce at follow-up evaluation, and 98 (55.7%) maintained the level obtained after the intervention. 

Within passive limb joints’ range of motion goals, two (7.1%) increased their GAS scores between post-intervention and follow-up evaluation sessions, 17 (60.8%) maintained post-intervention levels, and nine (32.1%) reduced their GAS score at follow-up evaluation. At T4, between goals related to functional motor abilities, 30 (23.8%) increased their GAS scores, 64 (50.8%) remained constant, and 32 (25.4%) reduced their scores. Three (20.0%) goals concerning hand functioning increased their GAS score between T3 and T4, and 13 (80.0%) remained stable. Of those related to general physical fitness, two (28.6%) goal scores were reduced at follow-up intervention, and five (71.4%) maintained the same GAS level. 

Among all 40 participants, at the end of the intervention phase (T3), one (2.5%) subject obtained a T-score lower than 40 due to the onset of epilepsy after the first month of program implementation. Despite parental attempts to adhere to the suggested program, the rate of daily seizures limited the possibility of implementing the activity program as planned. On the other hand, 19 (47.5%) participants obtained a T-score higher than 60, revealing that these individuals’ treatment potential has been underestimated. At follow-up evaluation, two (4.8%) subjects obtained a T-score lower than 40. For both, health problems were raised. One participant lost walking ability due to the onset of unspecified hip pain (facial expression of pain appeared when the hip was passively moved). The other participant started to present absence seizures that interrupted the learning process of walking and standing. No statistical difference was found comparing T-scores obtained at post-intervention (T3) and follow-up (T4) evaluations (*p* = 0.501). 

### 3.2. Motor Functioning

Participants’ motor functioning was assessed at each evaluation session throughout the RESMES. Descriptive statistics of participants’ results are collected in Table 3. The results of the conducted data analysis (Friedman test, Wilcoxon signed-rank test, and related effect sizes) are presented in Table 4.

Friedman test results showed a change across the evaluation sessions in the RESMES total score and all its subscales’ scores except for the Run subscale. Within the baseline phase (T1–T2), nine (22.5%) participants improved their total RESMES score, and one (2.5%) worsened it with an average change of −0.2 ± 1.1 points (range: +5–−4 points). No statistically significant difference emerged between RESMES score at T1 and T2. After the intervention phase (T2–T3), 34 (85.0%) participants showed a significant reduction in RESMES score (suggesting improvements in motor functioning), and no one (0%) increased it with an average change of −2.8 ± 2.5 points (range: 0–−10 points) and a large effect size (0.726). In the T3–T4 (wash-out) period, the average improvement in our group went back to the pre-intervention level with an average change of −0.3 ± 1.7 points (range: +3–−6 points). In the latter period, eleven (27.5%) subjects improved their score, and eight (20%) participants worsened their motor abilities. Moreover, the variation in the RESMES scores was similar in the baseline phase (∆T2–T1) and wash-out phase (∆T4–T3). The RESMES score change in the intervention phase (∆T3–T2) was more consistent, showing a statistical difference from the other two phases. The difference in the RESMES score changing velocities showed a large effect size between the baseline and intervention phases (0.635) and a moderate effect size between the intervention and follow-up phases (0.539). 

### 3.3. MBAR Physical Activity Level

The level of physical activity was assessed for all participants using the mBAR. Descriptive statistics of obtained results are collected in Table 3. Conducted analysis (Table 4) showed that the level of physical activity did not change between T1 and T2 (average change: 0.3 ± 4.5, range: +10–−18), significantly increased between T2 and T3 (average change: 9.0 ± 9.8, range: +28–−14) with large effect size (0.728), and reduced between T3 and T4 returning to pre-intervention level (average change: −7.6 ± 16.0, range: +33–−39) with a moderate effect size (0.470). Coherently, the average change in daily physical activity level showed a significant increase between the baseline and intervention phases with a large effect size (0.829) and a significant decrease between the intervention and wash-out phases again with a large effect size (0.718). 

### 3.4. Correlation between Variables

Results derived from correlation analysis are collected in Table 5.

A moderate positive relation was identified between the RARS and RESMES collected at T1, showing that participants with a higher RTT severity present lower motor functioning. Moreover, a moderate inverse relation was identified between the RESMES and mBAR score at T1, expressing that a higher motor functioning level is related to a higher daily level of physical activity. The improvement obtained at the RESMES in the intervention phase (∆T3–T2) showed a moderate inverse relation with RARS and RESMES scores collected at T1. These relations mean that participants with a more severe RTT manifestation or lower motor functioning improved more than individuals presenting less severe clinical presentation in the intervention phase. There were no correlations between RESMES scores improvement and participants’ age at T1. Regarding the level of rehabilitation goals achievement, no correlation was found between the GAS T-scores obtained at post-intervention (T3) and follow-up (T2) evaluations and any other investigated variable. There was no correlation between mBAR changes during the intervention phase and participants’ age, severity level, and motor functioning collected at baseline.

## 4. Discussion

The current paper describes the effect of a remotely supervised motor rehabilitation program carried out within the daily routine of girls and women with RTT. The obtained results strongly support the efficacy of such programs in improving the participants’ motor functional abilities and the achievement of rehabilitation goals confirming previous reports [13,21,47]. The present investigation involves the largest sample ever recruited in research regarding the motor treatment of individuals with RTT, providing supporting evidence of the validity of the proposed approach as an effective physical rehabilitation strategy for this population. Moreover, the current paper explored the effect of a structured cycle of remote supervision on motor function and physical activity level. 

The majority of the selected rehabilitation goals (82.4%) were achieved or overachieved at the end of the intervention phase. More precisely, 65.5% of these goal achievements exceed the researchers’ expectations, highlighting the efficacy of motor rehabilitation programs when applied to clients’ daily routines. The T-score data supported this statement, revealing an underestimation of the treatment potential for almost half of the sample (47.5%). Interestingly, the GAS scores indicate that functional physical goals (referred to gross and fine motor skills) were more likely to be achieved than those related to passive range of motion and general physical health. Even if only seven general physical-health-related goals were set, these results suggest that these goals are more challenging to be reached. The authors believe that improvements in motor functioning were more motivating for the participants’ parents and more easily maintained in practice. When a motor skill improves, the improvement is immediately recognizable by the client’s environment, which naturally prompts the sustenance of the new level of the motor skill, asking the person to use it within daily living situations. On the other hand, achievements related to general physical fitness (such as weight reduction) bring less observable or immediate improvements, thereby challenging the caregivers’ motivation to adhere to the tasks in the program that are strictly related to such achievements. Moreover, improvements in limb joint mobility frequently require specific maneuvers that could be difficult to perform by non-professionals (such as caregivers). Therefore, when planning rehabilitation programs, such as those presented here, all the goals (including those for physical fitness and joint mobility) should be pursued through functional activities that can be integrated into the participants’ daily lives. This consideration is in line with previous findings [13,21]. 

Furthermore, consideration should be made regarding goal achievements related to hand functioning, as 86.7% of these goals were achieved or overachieved. Due to the disorder’s characteristics, the hands of individuals with RTT are frequently left untreated [48]. However, there is scarce evidence regarding the efficacy of intensive programs in improving hand functioning in this population [49,50,51,52,53]. Pieces of evidence suggest that the hand functioning of people with RTT can be improved with repeated, individual, well-structured training [52,53]. Moreover, suggestions were made on the need to focus the training on functional skills that the participant can use in daily living situations [49,52]. The results of the present investigation support these statements and add to those that underline the need to consider manual function when planning a rehabilitation treatment for the population with RTT [49,52,53].

The functional motor enhancements obtained at the end of the treatment are confirmed by the RESMES results that showed a significant improvement in all the subscales (except the Run subscale) and the total score (with a large effect size). Moreover, no statistical difference was recognized when comparing the RESMES score obtained at T3 and T4. Therefore, the results suggest that the achieved rehabilitation goals could be maintained over time even when supervision is terminated. However, the RESMES and mBAR results highlight the impact of the supervision cycles. Within the treatment phase, when the participants’ caregivers are followed remotely, the RESMES improvements were more significant than during the baseline and wash-out phases. At the end of the treatment, the RESMES improvement velocity reduces, returning to the baseline level. Similarly, the daily physical activity level was significantly higher when the supervisions were active (intervention) than in the other phases. These results suggest the importance of following the caregivers carrying out such a program. However, a previous report [19] indicated that prolonged involvement in a supervised rehabilitation program might be hard to sustain for caregivers. Therefore, it is advisable to carry out these programs at three-month intervals, alternating periods of improvement with periods of recovery and consolidation of progress. Moreover, the observed improvement in the daily physical activity level could have been supported by the daily routine established by the provided programs and the involvement of participants’ parents in line with previous reports highlighting these aspects as facilitators of the promotion of light physical activities, such as standing and walking, in RTT [54] and other intellectual disabilities [55,56]. However, previous findings concerning the effect of similar activity programs on increasing the uptime of people with RTT have yielded conflicting results. Stahlhut et al., 2020 reported an increase in the uptime of girls with RTT after a 12-week program of enjoyable activities at home, school/day center, and community settings [57]. On the other hand, Downs et al., 2022 presented a multicenter randomized controlled study on the effects of a 12-week telehealth-supported physical activity program, reporting no difference between the change in the uptime of participants in the intervention and control group, evidencing positive intervention effect [58]. The differences in the obtained results can be explained by different perceptions of the caregivers carrying out the programs (reported to be perceived as “physically and psychologically draining” in Downs et al., 2022) or other impediments to progress, such as participants’ health problems. Therefore, the presented results related to improvement in physical activity should be interpreted cautiously.

The correlation analysis confirmed that higher severity of RTT presentation is associated with reduced motor functioning, in line with a previous finding [40]. However, no relation was found between the demographic variables and the levels of goal achievement (T-scores), indicating that neither severity level nor age affected the achievement of rehabilitation goals when these are individually set and targeted on the participants’ characteristics. Interestingly, the inverse correlations between the RESMES improvements within the intervention phase and the level of RTT severity and motor functioning collected at T0 suggest that more complex individuals with lower motor functioning are more likely to improve their motor skills when intensive, daily based, individualized activity programs are applied. These findings strongly support the need for providing such programs to all individuals with RTT across all ages and severity levels from a young age, in accordance with previous reports on such programs [13,21,23,59,60,61,62,63,64].

In light of the above-described results and considerations, the authors underline the need to provide the person with RTT with a lifelong, individualized, daily based, and professionally supervised rehabilitation path. The therapeutic intervention should be family-centered and constructed in strict cooperation with the person’s family to focus on significant goals and adequate support, enhancing adherence to the intervention.

This study presents some limitations. Although the recruited participants acted as controls for themselves, no control group was involved. Therefore, the gold standard randomized clinical trial design was not used, limiting the strength of the current study evidence. However, the implemented multiple baselines and wash-out phases prove that the implemented programs caused changes during the treatment phase. Moreover, the individualized nature of the current research could limit the reproduction of the results. Nevertheless, providing individualized treatment is mandatory in rehabilitation and even more important when facing a high variable pathology such as RTT presenting a wide range of clinical pictures. The authors provided information about how the programs and rehabilitation goals were planned and implemented to support clinicians constructing individualized programs for their clients. In addition, in the current study, the m-BAR was used to assess physical activity even though more reliable assessment tools were suggested (such as accelerometers) [65]. A further limitation could be represented by the different therapeutic regimens followed by the participants. However, the participants did not change their therapeutic regimen during any phase of the study, and the applied statistical analysis considered that the compared variables came from the same group, limiting the influence of the therapeutic regimen on the presented results. Finally, no data were collected on the participants’ adherence to the proposed programs, challenging the study’s internal validity. However, the temporal contingencies of the improvement in the outcome measures in the absence of other significant changes in the participants’ routine (including the educational and rehabilitative regimen) support the efficacy of the intervention. 

## 5. Conclusions

The proposed activity programs supported the achievement of therapeutic goals, improved gross and fine motor skills, and enhanced the daily physical activity level of girls and women with RTT across a wide range of ages and severity levels. Together with previous findings related to similar activity programs, the current paper claims the provision of similar interventions for all people with RTT and invites clinicians to establish such programs with their clients to maintain their higher level of motor functioning and physical activity fitness. The authors’ opinion is that the proposed intervention might also be beneficial to others with severe disabilities. Moreover, the current investigation highlighted the importance of remote supervision when establishing a home-based rehabilitation treatment with this population. Finally, the presented results indicate that functional motor goals (e.g., improving gross motor skills and hand functional performances) are more likely to be achieved within a home-based program. In contrast, goals related to improving joint range of motion and physical fitness could require more professional support. 

## Figures and Tables

**Table 1:** Participants’ ages and RTT severity levels measured at the first evaluation (T1).

*n* = 40	Age (Years)	RARS Scores						
		Cognition	Sensoriality	Motricity	Emotion	Autonomy	RTT Characteristics	Total
**Mean (SD)**	15.7 (9.7)	15.2 (3.9)	3.3 (1.1)	10.0 (2.6)	3.8 (1.2)	11.0 (1.7)	24.2 (3.8)	67.4 (10.0)
**Median**	13.3	15.3	3.0	10.0	3.5	11.8	24.0	67.8
**Range**	2.8–40.3	9.0–25.0	2.0–6.0	5.0–15.5	2.0–7.0	4.5–12.0	15.0–33.0	45.5–82.5

**Table 2:** Amount of goals for each area and related level of achievement at post-intervention (T3) and follow-up (T4) evaluation sessions.

Evaluation Session	Goal Area		Motor Function	Range of Motion	Hand Functioning	Physical Fitness	Total
	Amount (%)		126 (71.6%)	28 (15.9%)	15 (8.5%)	7 (4.0%)	176 (100%)
**T3**	**Gas scores**	**−2**	5 (4.1%)	0 (0%)	0 (0%)	1 (14.2%)	6 (3.4%)
		**−1**	14 (11.1%)	6 (21.4%)	2 (13.3%)	3 (42.9%)	25 (14.2%)
		**0**	39 (30.9%)	3 (10.7%)	7 (46.7%)	1 (14.2%)	50 (28.4%)
		**1**	43 (34.1%)	18 (64.3%)	2 (13.3%)	2 (28.6%)	65 (36.9%)
		**2**	25 (19.8%)	1 (3.6%)	4 (26.7%)	0 (0%)	30 (17.1%)
**T4**	**Gas scores**	**−2**	4 (3.2%)	3 (10.7%)	0 (0%)	2 (28.6%)	13 (7.4%)
		**−1**	20 (15.8%)	6 (21.4%)	2 (13.3%)	2 (28.6%)	38 (21.6%)
		**0**	39 (30.9%)	4 (14.4%)	5 (33.3%)	2 (28.6%)	52 (29.5%)
		**1**	36 (28.6%)	13 (46.4%)	3 (20.0%)	1 (14.2%)	55 (31.2%)
		**2**	27 (21.4%)	2 (7.1%)	5 (33.3%)	0 (0%)	34 (19.3%)

**Table 3:** RESMES and mBAR descriptive statistics for each evaluation session.

			Evaluation Session			
			T1	T2	T3	T4
**RESMES**	**Standing**	**Mean (SD)**	3.1 (3.9)	3.1 (3.9)	2.6 (3.4)	2.8 (3.6)
		**Median**	1	1	0	0
		**Range**	0–12	0–12	0–12	0–12
	**Sitting**	**Mean (SD)**	1.8 (3.2)	1.7 (3.1)	1.2 (2.3)	1.2 (2.1)
		**Median**	0	0	0	0
		**Range**	0–12	0–12	0–10	0–10
	**Transfer**	**Mean (SD)**	14.5 (7.6)	14.5 (7.6)	14.0 (7.4)	13.9 (7.5)
		**Median**	16	16	15	14.5
		**Range**	0–26	0–26	0–26	0–27
	**Walking**	**Mean (SD)**	7.9 (7.0)	7.9 (7.0)	7.2 (6.8)	7.0 (6.9)
		**Median**	6	6	5	5
		**Range**	0–18	0–18	0–18	0–18
	**Run**	**Mean (SD)**	3.8 (0.8)	3.8 (0.8)	3.8 (0.8)	3.7 (1.0)
		**Median**	4	4	4	4
		**Range**	0–4	0–4	0–4	0–4
	**Stairs**	**Mean (SD)**	6.5 (2.0)	6.4 (2.0)	5.9 (2.3)	5.9 (2.2)
		**Median**	7	7	6	6
		**Range**	0–8	0–8	0–8	0–8
	**Total**	**Mean (SD)**	37.6 (21.6)	37.4 (21.6)	34.6 (20.5)	34.6 (20.5)
		**Median**	33	32.5	29	29
		**Range**	0–80	0–80	0–74	0–74
**mBAR**		**Mean (SD)**	98.2 (27.5)	98.5 (26.1)	107.5 (26.7)	100.0 (27.5)
		**Median**	100	101	107	100
		**Range**	52–168	51–150	51–167	51–155

**Table 4:** Results from the Friedman and Wilcoxon signed-rank test analyses comparing participants’ scores obtained at RESMES and mBAR at all four evaluation points and the changes that occurred during each phase of the current project.

		RESMES							mBAR
		Standing	Sitting	Transfer	Walking	Run	Stairs	Total	
**Friedman test**		0.002 * (0.127)	0.002 * (0.124)	˂0.001 * (0.225)	˂0.001 * (0.303)	0.733	˂0.001 * (0.304)	˂0.001 * (0.753) ^§§^	˂0.001 * (0.205)
**Wilcoxon signed-rank test**	**T1 vs. T2**	1.000	0.180	0.317	0.480	1.000	0.317	0.059	0.084
	**T1 vs. T3**	0.017 * (0.101) ↑	0.017 * (0.034) ↑	0.002 * (0.090) ↑	0.001 * (0.213) ↑	0.317	0.001 * (0.111) ↑	˂0.001 * (0.683) ^§§^↑	˂0.001 * (0.720) ^§§^↑
	**T1 vs. T4**	0.060	0.027 * (0.045) ↑	0.001 * (0.159) ↑	0.001 * (0.265)	0.414	0.001 * (0.111) ↑	˂0.001 * (0.768) ^§§^↑	0.380
	**T2 vs. T3**	0.009 * (0.100) ↑	0.027 * (0.026) ↑	0.002 * (0.090) ↑	˂0.001 * (0.239) ↑	0.317	0.001 * (0.111) ↑	˂0.001 * (0.726) ^§§^↑	˂0.001 * (0.728) ^§§^↑
	**T2 vs. T4**	0.076	0.049 * (0.034) ↑	0.002 * (0.147) ↑	0.001 * (0.263) ↑	0.414	0.001 * (0.111) ↑	˂0.001 * (0.726) ^§§^↑	0.591
	**T3 vs. T4**	0.210	0.581	0.479	0.341	0.564	0.480	0.461	0.010 * (0.470) ^§^↓
	**∆T2–T1** **vs.** **∆T3–T2**	0.009 * (0.100) ↑	0.107	0.003 * (0.080) ↑	˂0.001 * (0.238) ↑	0.317	0.001 * (0.111) ↑	˂0.001 * (0.635) ^§§^↑	˂0.001 * (0.829) ^§§^↑
	**∆T3–T2** **vs.** **∆T4–T3**	0.008 * (0.123) ↓	0.028 * (0.038) ↓	0.070	0.025 * (0.155) ↓	1.000	0.005 * (0.107) ↓	˂0.001 * (0.539) ^§^↓	˂0.001 * (0.718) ^§§^↓
	**∆T2–T1** **vs.** **∆T4–T3**	0.174	0.671	0.599	0.446	0.564	0.408	0.614	0.009 * (0.440) ^§^↑

The value in each cell represents the comparison’s *p*-value (comparison’s effect size). The effect size was reported if the comparison was statistically significant (*p*-value ˂ 0.05). ∆: arithmetical difference between the scores collected at two different evaluation sessions; *: *p*-value ˂ 0.05; ^§^: medium effect size; ^§§^: large effect size; ↑: the difference represents an improvement compared to the previous evaluation session or study phase; ↓: the difference represents an improvement compared to the previous evaluation session or study phase.

**Table 5:** Levels of significance, *p*-value, and correlation coefficients (ρ—in parenthesis) of the explored relations between variables.

	Participants Age	RARS T1	RESMES T1	mBAR T1
**Participants age**	/	0.650 (−0.074)	0.168 (0.222)	0.102 (−0.262)
**RARS T1**	0.650 (−0.074)	/	0.019 (0.368) *	0.083 (−0.277)
**RESMES T1**	0.168 (0.222)	0.019 (0.368) *	/	˂0.001 (−0.695) *
**mBAR T1**	0.102 (−0.262)	0.083 (−0.277)	˂0.001 (−0.695) *	/
**RESMES ∆T2–T1**	0.121 (0.249)	0.380 (−0.142)	0.884 (0.024)	0.871 (0.026)
**RESMES ∆T3–T2**	0.640 (−0.076)	0.005 (−0.440) *	˂0.001 (−0.531) *	0.116 (0.253)
**RESMES ∆T4–T3**	0.984 (0.003)	0.482 (0.115)	0.578 (0.091)	0.910 (0.018)
**mBAR ∆T2–T1**	0.791 (0.043)	0.737 (−0.055)	0.910 (−0.019)	0.122 (−0.249)
**mBAR ∆T3–T2**	0.217 (0.199)	0.495 (−0.111)	0.920 (0.016)	0.712 (−0.06)
**mBAR ∆T4–T3**	0.611 (−0.083)	0.924 (0.016)	0.414 (−0.133)	0.359 (−0.149)
**GAS T-score T3**	0.318 (0.162)	0.347 (−0.153)	0.916 (−0.017)	0.458 (−0.121)
**GAS T-score T4**	0.414 (0.133)	0.412 (−0.133)	0.450 (0.123)	0.117 (−0.252)

*: *p*-value ˂ 0.05.

## Data Availability

The dataset presented in this paper can be requested from the corresponding author.

## Supplementary Materials

The following supporting information can be downloaded at: https://www.mdpi.com/article/10.3390/ijerph20010659/s1, Table S1: Individual age, MECP2 gene mutation, RARS total and subscales scores at T1, and RESMES total and subscales scores at T1; Table S2: Pre-intervention, post-intervention (T3), and follow-up (T4) GAS levels and T-scores for each goal and each participant.
