# Supplementary material for: Individualized Remotely Supervised Motor Activity Programs Promote Rehabilitation Goal Achievement, Motor Functioning, and Physical Activity of People with Rett Syndrome—A Single-Cohort Study
Source: Int J Environ Res Public Health. 2022 Dec 30;20(1):659. doi: 10.3390/ijerph20010659 (PMC9819180; doi:10.3390/ijerph20010659)
Supplement: Supplementary file 1 [file ijerph-20-00659-s001.zip › ijerph-2057606-supplementary/Table S1.pdf]

Supplementary material 1. Individual age, MECP2 gene mutation, RARS total and subscales scores at T1, and RESMES total and subscales scores at T1.

Pz.	Age at baseline	MECP2 gene Mutation	RARS T1							RESMES T1						
			Cognition	Sensoriality	Motricity	Emotion	Autonomy	Rett characteristics	Total	Standing	Sitting	Transfer	Walking	Run	Stairs	Total
1	13.3	R168X	16	4	14	3	12	24	73	3	4	20	12	4	7	50
2	38.2	MECP2 mutation not otherwise specified	19	3	15.5	5.5	12	27.5	82.5	12	12	26	18	4	8	80
3	11.5	c.916 C>T P.Arg306cys	12.5	4	13	4	11.5	29.5	74.5	4	9	18	14	4	8	57
4	22.0	P152R	12.5	3	9.5	2.5	9.5	24.5	61.5	1	0	16	6	4	6	33
5	27.7	pro225arg	15.5	5.5	9.5	4	11	22	67.5	2	0	18	7	4	6	37
6	13.1	c.468 C>G	15	3	13	3	12	25	71	6	1	24	17	4	8	60
7	18.1	del. Exon 3-4	20	2	9	5	12	23	71	0	0	12	6	4	7	29
8	17.2	c.502 C>T P.Arg168Stop	10.5	2	5.5	2.5	10	19.5	50	0	0	13	2	4	6	25
9	21.4	del. Exon 3-4	14.5	3	12	4	12	22.5	68	4	4	23	16	4	8	59
10	16.9	c. 1160_1166 Del ccccacc; p.P387fsX406	11	4.5	5.5	2	6.5	23	52.5	0	0	2	0	2	2	6
11	5.4	c.473 C>T p.thr158met	10.5	2.5	5	3	11	24	56	0	0	2	0	2	6	10
12	18.7	P152R c.455 C>G	21	5	13	4.5	12	25.5	81	4	8	24	17	4	8	65
13	12.8	c.916 C>T	18.5	5	10.5	5	11	27	77	0	0	7	0	4	4	15
14	12.3	P.Arg133cys	13.5	2.5	7	3	4.5	15	45.5	0	0	0	0	4	2	6
15	6.5	c.473 C>T p.thr158met	17.5	4	9	4	11.5	19	65	1	0	20	2	4	8	35
16	13.3	P133C	15.5	3	10.5	4	10.5	29.5	73	0	0	13	5	4	7	29

17	10.3	c.763 C>T	15	2	8.5	3	12	21.5	62	0	0	16	5	4	6	31
18	31.6	R255	10.5	3.5	12.5	3.5	12	25	67	11	0	22	18	4	8	63
19	4.2	c. 808 C>T p. Arg270	19	2.5	5.5	5	10	21.5	64.5	0	0	0	0	0	0	0
20	2.8	del. Exon 4	23	5	10.5	4.5	12	27.5	82.5	3	3	17	11	4	8	46
21	3.7	p. Arg255	25	4.5	7	6	11	26	79.5	0	0	7	2	4	7	20
22	40.3	MECP2 mutation not otherwise specified	13.5	2	8.5	3	7	20.5	54.5	0	0	16	6	4	5	31
23	16.7	T158M	11.5	2	13	3	11	25.5	66	11	5	24	18	4	8	70
24	5.3	c. 316 C>T p. R106W	18.5	4	8	3.5	12	25.5	71.5	7	0	17	15	4	8	51
25	14.4	p. R106W	13.5	4	13	3	11.5	21	66	0	0	8	2	4	6	20
26	12.7	c. 473 C>T	10.5	3	10	2.5	9	19.5	54.5	6	3	19	15	4	8	55
27	29.2	del. Exon 3	18	6	10	7	11.5	27	79.5	0	0	15	0	4	4	23
28	13.6	c. 1063-1326 del. 264 + insTGCG	11	2	10.5	2	12	24	61.5	0	0	7	0	4	6	17
29	5.8	c. 965 C>T p.Pro322Leu	11.5	2	6.5	2.5	12	23.5	58	0	0	5	0	4	5	14
30	9.5	c. 808 C>T	17.5	2	14	5.5	12	28.5	79.5	9	11	26	15	4	8	73
31	3.8	c.880 C>T p. Arg294	14	5	10	2.5	10	27.5	69	4	0	10	6	4	8	32
32	14.1	p.Arg306Cys	19.5	3	8	2.5	11.5	20	64.5	0	0	9	2	3	5	20
33	24.6	T158M	15.5	3	10	6	12	21.5	68	8	4	22	17	4	8	63
34	12.9	c. 882 C>T p. R294X	16.5	3	13	4	12	33	81.5	8	1	21	15	4	8	57
35	38.3	T158M	9.5	2	8.5	3	9	23.5	55.5	0	0	7	2	4	3	16

36	5.5	c.763 C>T p. Arg255	16.5	3.5	10	5.5	12	31.5	73	0	0	9	2	4	8	23
37	22.1	MECP2 mutation not otherwise specified	19.5	4	10.5	4	12	25.5	75.5	2	2	11	8	4	8	35
38	13.3	c. 763 C>T	16	3.5	11	4	12	27	73.5	0	0	9	0	4	6	19
39	14.8	del. Exon 3-4	11	3.5	11.5	3.5	12	22.5	64	10	4	25	18	4	8	69
40	8.8	c. 502 C>T p.Arg168X	9	2	9.5	2	12	17	51.5	9	0	19	18	4	8	58

Abbreviation list: Pt. = Participant; RARS = Rett Assessment Rating Scale; RESMES = Rett Syndrome Motor Evaluation Scale.
