# Supplementary material for: Individualized Remotely Supervised Motor Activity Programs Promote Rehabilitation Goal Achievement, Motor Functioning, and Physical Activity of People with Rett Syndrome—A Single-Cohort Study
Source: Int J Environ Res Public Health. 2022 Dec 30;20(1):659. doi: 10.3390/ijerph20010659 (PMC9819180; doi:10.3390/ijerph20010659)
Supplement: Supplementary file 1 [file ijerph-20-00659-s001.zip › ijerph-2057606-supplementary/Table S2.pdf]

Table S2. Pre-intervention, post-intervention (T3), and follow-up (T4) GAS levels and related T-scores for each goal and each participant. This supplementary material is meant to help clinicians working with individuals with RTT to find adequate therapeutic goals for their clients. The reference age, RTT severity level, and motor functioning level of each participant are available in Supplementary material 1.

Pt.	Goal area	Pre-intervention (T2) level	Level reached at post-intervention (T3)	GAS score at T3	T-score at T3	Level reached at follow-up (T4)	GAS score at T4	T-score at T4
1	Motor function	Can walk for 8 steps independently	Can walk for 15 steps independently	0	60.6	Can walk for 25 steps independently	+1	58.5
	Motor function	Can sit on an air cushion for 30''	Can sit on an air cushion for 2'	+1		Can sit on an air cushion for 1'	0	
	Motor function	Can stand independently for 15''	Can stand independently for 1'	+1		Can stand independently for 1'	+1	
2	Range of motion	Hands 1st interphalangeal extension ROM (30° bilat)	Improvement of 10°	+1	60.0	Improvement of 10°	+1	67.5
	Range of motion	Shoulder flexion ROM (115° Rt - 90° Lt)	Improvement of 5°	0		Improvement of 5°	0	
	Range of motion	Shoulder abduction ROM (85° Rt - 40° Lt)	Improvement of 10°	+1		Improvement of 10°	+1	
	Motor function	Can sit unsupported for 30''	Can sit unsupported for 2'	+1		Can sit unsupported <5'	+2	
3	Motor function	Can walk in the pool for 30 min without a flotation device, and the water at the chest level	Can walk in the pool for 20 min without a flotation device, and the water at the pelvis level	+1	59.6	Can walk in the pool for >30 min without a flotation device, and the water at the pelvis level	+2	62.8
	Physical fitness	Weight: 54 Kg	No weight variation	-1		No weight variation	-1	
	Motor function	Can sit for 5'' without back support with strong support to the pelvis	Can sit for 5'' without back support independently	+2		Can sit for 5'' without back support independently	+2	
	Motor function	Can sit without back support for 10''	Can sit without back support up to 2'	+1		Can sit without back support for more than 2'	+2	
4	Motor function	Can climb a stair with strong support given to both arms from the front	Can climb a stair with support given to one hand and the	+1	61.1	Can climb a stair with support given to one hand and the other hand maintained on the rail	+1	67.4

			other hand maintained on the rail					
	Motor function	Can descend a stair with strong support given to both arms from the front	Can descend a stair with strong support given to both arms from the front	-1		Can descend a stair with support given to one wrist and the other hand maintained on the rail	+1	
	Motor function	Amount of time she needs support to prevent falls while walking independently on uneven terrain in 10 min (field with high grass) (7 times) with a safety belt	Amount of time she needs support to prevent falls while walking independently on uneven terrain in 10 min (field with high grass) (1 time) with a safety belt	+1		Amount of time she needs support to prevent falls while walking independently on uneven terrain in 10 min (field with high grass) (1 time) with a safety belt	+1	
	Range of motion	Left shoulder flexion ROM	Improvement of 10°	+1		Improvement of 10°	+1	
	Range of motion	Left shoulder abduction ROM	Improvement of 10°	+1		Improvement of 10°	+1	
	Range of motion	Right shoulder flexion ROM	Improvement of 10°	+1		Improvement of 10°	+1	
	Range of motion	Right shoulder abduction ROM	Improvement of 10°	+1		Improvement of 10°	+1	
5	Physical fitness	Weight: 64.3 Kg	No weight variation	-1	51.4	No weight variation	-1	61.0
	Motor function	Can walk for 5 steps independently	Can walk for 5 steps independently	-1		Can walk for 10 steps independently	0	
	Motor function	Can walk 10 steps with support given to one hand	Can walk 10 steps with a light touch (one finger) on her shoulder	+1		Can walk 10 steps independently	+2	
	Motor function	Can stand for 1' independently	Can stand for 5' independently	+1		Can stand for >5' independently	+2	
	Range of motion	Fixed head flexion to the left for 24°	Improvement of 10°	+1		Improvement of 10°	+1	
6	Motor function	Can sit on the floor with crossed legs for 2'	Can sit on the floor with crossed legs for 10'	+1	59.0	Can sit on the floor with crossed legs for 10'	+1	60.5
	Motor function	Can stand for 10" independently, holding the rail by herself	Can stand for 10" independently, holding the rail by herself	-1		Can stand for 10" independently, holding the rail by herself	-1	

	Range of motion	Head left flexion limited to 15°	Improvement of 10°	+1		Improvement of 10°	+1	
	Range of motion	Head left rotation limited to midline	Full ROM	+2		Full ROM	+2	
	Range of motion	Hip extension limited to -20°	Improvement of 5°	0		Hip extension limited for -20°	-1	
7	Motor function	Amount of time she needs support to prevent falls while walking independently on uneven terrain in 10 min (field with high grass) (5 times) with a safety belt	Never needs support to prevent falls with a safety belt	+2	81.9	Never needs support to prevent falls with a safety belt	+2	75.6
	Motor function	Can walk 10 steps independently with a safety belt	Can walk >50 steps independently with a safety belt	+2		Can walk >50 steps independently with a safety belt	+2	
	Motor function	Can climb a stair with full support given to the trunk from behind with elastic bandages	Can climb a stair with support given to one hand and the other hand maintained on the rail with elastic bandages	+2		Can climb a stair with support given to both wrists with elastic bandages	+1	
	Motor function	Can descend a stair with strong support given to the trunk from behind with elastic bandages	Can descend a stair with support given to one wrist and the other hand maintained on the rail with elastic bandages	+2		Can descend a stair with strong support given to both wrists from the front with elastic bandages	+1	
8	Motor function	Amount of time she needs support to prevent falls while walking independently on uneven terrain in 10 min (field with high grass) (5 times)	Never needs support to prevent falls	+2	50.0	Never needs support to prevent falls	+2	65.3
	Motor function	Can walk up a mild slope with support given to one hand	Can walk up a mild slope with support given to one sleeve	0		Can walk up a mild slope with support given to one sleeve	0	
	Motor function	Cannot walk up a slope independently	Cannot walk up a slope independently	-2		Can walk 5 steps up a slope independently	-1	

	Motor function	Can walk down a mild slope with support given to one hand	Can walk down a mild slope independently with a safety belt	+1		Can walk down a mild slope independently with a safety belt	+1	
	Motor function	Can walk 5 steps down a slope independently	Cannot walk down a slope independently	-2		Can walk up to 20 steps down a slope independently	+1	
9	Physical fitness	Pre-intervention bone density	Pre-intervention bone density	-1	48.8	Pre-intervention bone density	-1	52.4
	Motor function	Can climb a stair with strong support given to both arms from the front and elastic bandages for leg bending	Can climb a stair with strong support given to both arms from the front and elastic bandages for leg bending	-1		Can climb a stair with support given to both wrists and elastic bandages for leg bending	0	
	Motor function	Can descend the stair with strong support given from the front with one hand under her armpit and one hand holding her arm and elastic bandages for leg bending	Can descend the stair with strong support given from the front to both arms and elastic bandages for leg bending	0		Can descend the stair with strong support given from the front to both arms and elastic bandages for leg bending	0	
	Motor function	Can stand up with slight support given to wrists	Can stand up with slight support given to one wrist	0		Can stand up with slight support given to one wrist	0	
	Motor function	Can sit on a chair with intense sensory input given to the head and the pelvis (hips flexion) when the back of her knees touches the chair	Can sit on a chair with intense sensory input given to the head and the pelvis (hips flexion) when the back of her knees touches the chair	-1		Can sit on a chair with light sensory input given to the head and the pelvis (hips flexion) when the back of her knees touches the chair	0	
	Range of motion	Elbow extension: -60°	Elbow extension: -60°	-1		Deterioration of at least 10°	-2	
	Motor function	Can stand for 1 to 2 min by herself, leaning with her hand on a piece of furniture	Can stand for >5 min by herself, leaning with her hand on a piece of furniture	+2		Can stand for >5 min by herself, leaning with her hand on a piece of furniture	+2	
10	Motor function	Toe walking for <70% of the time when without splints	Toe walking for <20% of the time when without splints	+1	67.0	Toe walking for <50% of the time when without splints	0	67.0
	Hand functioning	Participate in table setting, putting one object on the table	Participate in table setting, putting more than four objects on the table	+2		Participate in table setting, putting more than four objects on the table	+2	

	Motor function	Can run independently for 10-20 steps	Can run independently for 20-30 steps	0		Can run independently for 30-40 steps	+1	
11	Motor function	Toe walking for <70% of the time when without splints	Toe walking for <50% of the time when without splints	0	57.0	Toe walking for <50% of the time when without splints	0	55.6
	Range of motion	Right ankle dorsal flexion at +3°	Improvement of 10°	+1		Improvement of 5°	0	
	Range of motion	Left ankle dorsiflexion at +2°	Improvement of 5°	0		Left ankle dorsiflexion at +2°	-1	
	Motor function	Can climb a stair independently with the hand on the rail, under strict supervision, with both feet on each step	Can climb a stair independently with the hand on the rail without supervision, with both feet on each step	0		Can climb a stair independently without supervision, with both feet on each step	+1	
	Motor function	Can descend a stair with support given to one hand and the other hand maintained on the rail	Can descend a stair independently, holding the rail with close supervision	+1		Can descend a stair independently, holding the rail with close supervision	+1	
12	Motor function	Can sit on a chair with intense sensory input given to the head and the pelvis (hips flexion) when the back of her knees touches the chair	Can sit on a chair with intense sensory input given to the head and the pelvis (hips flexion) when the back of her knees touches the chair	-1	43.2	Can sit on a chair with intense sensory input given to the head and the pelvis (hips flexion) when the back of her knees touches the chair	-1	41.5
	Motor function	Can stand up with slight support given to wrists	Can stand up with slight support given to one wrist	0		Can stand up with slight support given to one wrist	0	
	Range of motion	No worsening in both shoulders flexion	Improvement of 10° (both shoulders)	+1		ROM shoulder flexion (worsening in the left shoulder in follow-up evaluation)	-2	
	Range of motion	No worsening in both shoulders abduction	No worsening in both shoulders abduction	-1		ROM shoulder abduction (worsening in the left shoulder in follow-up evaluation)	-2	
	Range of motion	No worsening in both elbow extension	Improvement of 10° (both elbows)	+1		Improvement of 10° (both elbows)	+1	
	Motor function	Can sit independently on a stool in a vestibular plate for 1'	Can sit independently on a stool in a vestibular plate for 1'	-1		Can sit independently on a stool in a vestibular plate for 2'	0	

13	Motor function	Can climb the stair with support to one hand and the other hand on the rail in 1'	Can climb the stair held by one sleeve in 30"	+1	56.7	Can climb the stair with support to one hand and the other hand on the rail in 30"	0	43.3
	Motor function	Can descend the stair with support to one forearm and the other hand passively moved on the rail in 2'	Can descend the stair with support to one forearm and the other hand passively moved on the rail in 1'	0		Can descend the stair with support to one forearm and the other hand passively moved on the rail in 2'	-1	
14	Hand functioning	Can wear the T-shirt with the help of an adult	Can wear a T-shirt (head and one sleeve)	+1	75.0	Can wear tha-shirt (head and one sleeve)	+1	75.0
	Hand functioning	Can take the T-shirt off with the help of an adult	Can take the T-shirt off independently	+2		Can take the T-shirt off independently	+2	
	Hand functioning	Participate in table setting, putting one object on the table	Participate in table setting, putting more than four objects on the table	+2		Participate in table setting, putting more than four objects on the table	+2	
15	Motor function	Can stand up with slight support given to wrists	Can stand up with slight support given to wrists	-1	30.4	Can stand up with slight support given to one wrist and quick sensory input to the knee	0	52.8
	Motor function	Can sit on a chair with intense sensory input given to the head and the pelvis (hips flexion) when the back of her knees touches the chair	Can sit on a chair with light sensory input given to the head and the pelvis (hips flexion) when the back of her knees touches the chair	0		Can sit on a chair with light sensory input given to the head and the pelvis (hips flexion) when the back of her knees touches the chair	0	
	Motor function	Can stand independently for 2'	Can stand independently for less than 1'	-2		Can stand independently for 3'	0	
	Motor function	Can walk for 20 steps independently	Can walk for 10 steps independently	-2		Can walk for 30 steps independently	0	
	Motor function	Can sit in a vestibular plate in asymmetrical sitting for 1' with 2 falls	Can sit in a vestibular plate in asymmetrical sitting for 1' with 1 fall	0		Can sit in a vestibular plate in asymmetrical sitting for 1' with 0 fall	+1	
16	Motor function	Can climb a stair with strong support given to both wrists	Can climb a stair with support given to one sleeve and the	+1	62.4	Can climb a stair with support given to one sleeve and the	+1	62.4

			other hand maintained on the rail			other hand maintained on the rail		
	Motor function	Can descend a stair with strong support given to both arms from the front	Can descend a stair with support given to one wrist and the other hand maintained on the rail	+1		Can descend a stair with support given to one wrist and the other hand maintained on the rail	+1	
	Motor function	Can walk for 20 minutes before refusing to continue	Can walk for 45 minutes before refusing to continue	+1		Can walk for 45 minutes before refusing to continue	+1	
17	Motor function	Can stand up from a chair with light support given to both wrists	Can stand up from a chair with light support given to one wrist	0	56.7	Can stand up from a chair with light support given to one wrist	0	63.4
	Motor function	Can sit on a chair with intense sensory input given to the head and the pelvis (hips flexion) when the back of her knees touches the chair	Can sit on a chair with light sensory input given to the pelvis (hips flexion) when the back of her knees touches the chair	+1		Can sit on a chair independently when the back of her knees touches the chair	+2	
18	Range of motion	No knee flexion worsening	No knee flexion worsening	-1	64.9	No knee flexion worsening	-1	68.2
	Motor function	Can sit on the wedge without support to the back and the feet for <4 minutes	Can sit on the wedge without support to the back and the feet for >20 minutes (no time limit)	+2		Can sit on the wedge without support to the back and the feet for >20 minutes (no time limit)	+2	
	Motor function	Can walk for 2 minutes on the treadmill at 1km\h	Can walk for 10 minutes on the treadmill at 1km\h	+1		Can walk for 10 minutes on the treadmill at 1km\h	+1	
	Motor function	Can walk for 2 minutes on the treadmill at 1km\h, pulse at 120 bpm	Can walk for 2 minutes on the treadmill at 1km\h, pulse at 100 bpm	+1		Can walk for 2 minutes on the treadmill at 1km\h, pulse at 85 bpm	+2	
	Motor function	Can walk for 2 minutes on the treadmill at 1km\h	Can walk for 2 minutes on the treadmill at 1,5 km\h	0		Can walk for 2 minutes on the treadmill at 1,5 km\h	0	
19	Hand functioning	Can participate in the movements to wear the t-shirt inserting both arms in the sleeves	Can participate in the movements to wear the t-shirt placed on her head, inserting the head independently and both arms in the sleeves with	0	58.7	Can participate in the movements to wear the t-shirt placed on her head, inserting the head and both arms independently	+1	61.3

			little help (mother enlarged the t-shirt lower flap)					
	Hand functioning	Can participate in the movements to take off the t-shirt raising both arms	Can take off the t-shirt after the caregiver has taken off both arms	0		Can take off the t-shirt after the caregiver has taken off both arms	0	
	Hand functioning	Can participate in the movements to take the pants up with complete physical guidance	Can take the pants up independently to the pelvis level	+1		Can take the pants up independently	+2	
	Hand functioning	Can participate in the movements to take the pants down with complete physical guidance	Can take the pants down independently	+2		Can take the pants down independently	+2	
	Hand functioning	Can participate in the movements to take up the zip of the jacket with complete physical guidance	Can take the zip up when the jacket bottom flap is held by the caregiver and with easier adapted zip	0		Can take the zip up when the jacket bottom flap is held by the caregiver and with easier adapted zip	0	
	Hand functioning	Can participate in the movements to take down the zip of the jacket with complete physical guidance	Can take the zip down when the jacket bottom flap is held by the caregiver and with easier adapted zip	0		Can take the zip down when the jacket bottom flap is held by the caregiver and with easier adapted zip	0	
	Physical fitness	Can imitate at least one targets movement	Can imitate at least three targets movements	+1		Can imitate at least two targets movements	0	
	Physical fitness	Pee in the toilette at least 25% of the daily toilette times	Pee in the toilette at least 25% of the daily toilette times	-1		Pee in the toilette at least 25% of the daily toilette times	-1	
20	Motor function	Can walk >11 steps supported by two hands	Can walk >11 steps independently with close supervision	+1	53.8	Can walk >11 steps independently with close supervision	+1	53.8
	Motor function	Can walk at least 10 steps independently with close supervision	Can walk at least 50 steps independently with close supervision	+2		Can walk at least 50 steps independently with close supervision	+2	

	Motor function	Can stand for 1 minute supported by both arms	Can stand for 1 minute supported by one arm	0		Can stand for 1 minute supported by both arms	-1	
	Motor function	Can stand independently for 20 sec	Can't stand independently even for a few seconds	-2		Can stand independently for 20 sec	-1	
	Hand functioning	Can eat a piece of food with her hand with complete physical guidance (hand on hand)	Can eat a piece of food with her hand with partial physical guidance (hand on the wrist)	0		Can eat a piece of food with her hand with partial physical guidance (hand on the wrist)	0	
21	Hand functioning	Can eat a piece of food with her hand with complete physical guidance (hand on hand)	Can eat a piece of food with her hand with partial physical guidance (hand on the wrist)	0	54.2	Can eat a piece of food with her hand with partial physical guidance (hand on the arm)	+1	62.7
	Hand functioning	Able to maintain the bottle with both hands while drinking with complete physical guidance (hand-on-hand)	Able to maintain the bottle with both hands while drinking with partial physical guidance (hand on the wrist)	0		Able to maintain the bottle with both hands while drinking with partial physical guidance (hand on the wrist)	0	
	Motor function	Can walk independently on the lawn for <1 minute without falling	Can walk independently on the lawn for <5 minutes without falling	+1		Can walk independently on the lawn for <10 minutes without falling	+2	
	Motor function	Can climb the stairs with strong support given to both arms and elastic bandages to both knees	Can climb the stairs with strong support given to both arms	0		Can climb the stairs with strong support given to one arm	+1	
	Motor function	Can descend the stairs with strong support given to both arms and elastic bandages to both knees	Can descend the stairs with strong support given to both arms	0		Can descend the stairs with strong support given to both arms	0	
	Motor function	Can participate in the movement of standing up from the floor with strong support given to the trunk	Can participate in the movement of standing up from the floor with strong support given to both wrists	0		Can participate in the movement of standing up from the floor with strong support given to both wrists	0	
22	Motor function	Can climb the stair supported by one person in front of her to both wrists	Can climb the stair supported by one person in front of her holding one wrist and the other hand on the rail	0	54.7	Can climb the stair supported by one person in front of her holding one hand on the rail	+1	52.4

	Motor function	Can descend the stair supported by one person in front of her to both wrists	Can descending the stair supported by one person in front of her holding one hand on the rail	+1		Can descending the stair supported by one person in front of her holding one hand on the rail	+1	
	Motor function	Can assist the movement of standing up from a high chair (half flexed hip, 45°) when pulled from one wrist and with the chair gently tilted forward	Can assist the movement of standing up from a high chair (half flexed hip, 45°) when pulled from one wrist and with the chair gently tilted forward	-1		Can assist the movement of standing up from a high chair (half flexed hip, 45°) when pulled from one wrist and with the chair gently tilted forward	-1	
	Motor function	Can sit on a chair from standing with strong sensory input given to the pelvis	Can sit on a chair independently with verbal command when the chair is behind her	+1		Can sit on a chair from standing with gentle sensory input given to the pelvis	0	
	Range of motion	no deterioration	Improvement of 10°	+1		Improvement of 10°	+1	
23	Motor function	Can sit asymmetrically on the arm of the couch for <1 minute	Can sit asymmetrically on the arm of the couch for at least 20 minutes	+2	70.0	Can sit asymmetrically on the arm of the couch for at least 20 minutes	+2	57.5
	Motor function	Can stand holding support in front of her with both hands for at least 2 minutes	Can stand holding support in front of her with both hands for at least 5 minutes	0		Can stand holding support in front of her with both hands for at least 2 minutes	-1	
	Motor function	Can stand for 2 minutes supported by both arms from the front	Can stand for 2 minutes supported by both wrists from the front	+1		Can stand for 2 minutes supported by both arms from the front	-1	
	Motor function	Can walk 10 steps with full support from behind and help to move the legs forward	Can walk 10 steps with strong support given from the front to both arms	+1		Can walk 10 steps with full support from behind and help to move the legs forward	-1	
24	Motor function	Can sit on a chair from standing with light support given to head and pelvis and with feet maintained in position	Can sit on a chair from standing with light support given to the pelvis and with feet maintained in position	0	47.5	Can sit on a chair from standing with light support given to the pelvis and with feet maintained in position	0	51.2

	Motor function	Can stand independently leaning on the wall with the back for 45''	Can stand independently leaning on the wall with the back for 1'	0		Can stand independently leaning on the wall with the back for 1'	-1	
	Motor function	Can walk 20 steps supported by two wrists	Can walk 30 steps supported by two wrists	0		Can walk 30 steps supported by two wrists	+2	
	Motor function	Can walk 10 steps supported by two hands	Can walk 10 steps supported by two hands	-1		Can walk 10 steps supported by two hands	-1	
	Motor function	Can sit on a vestibular plate with crossed legs on a pillow for 20'	Can sit on a vestibular plate with crossed legs on a pillow for 30'	0		Can sit on a vestibular plate with crossed legs on a pillow for 30'	0	
	Motor function	Can sit on a vestibular plate with crossed legs on two pillows (10cm) for 10'	Can sit on a vestibular plate with crossed legs on a stool (34cm) for 10'	+1		Can sit on a vestibular plate with crossed legs on a stool (34cm) for 10'	+1	
25	Motor function	Can climb stairs with strong support given to both wrists	Can climb stairs with support given to one wrist	0	43.3	Can climb stairs with support given to one wrist	0	43.3
	Motor function	Can descend stairs with strong support given to both wrists from the front	Can descend stairs with strong support given to both wrists from the front	-1		Can descend stairs with strong support given to both wrists from the front	-1	
26	Motor function	Can stand for less than 1,5' supported by two wrists	Can stand for less than 10' supported by two wrists	+2	71.9	Can stand for less than 5' supported by two wrists	+1	71.9
	Motor function	Can stand for less than 1' supported by two hands	Can stand for less than 1' supported by two sleeves	+1		Can stand for less than 1' supported by two sleeves	+1	
	Motor function	Can walk 10 steps with strong trunk support from behind	Can walk 30 steps with strong trunk support from behind	+1		Can walk 40 steps with strong trunk support from behind	+2	
	Motor function	Can walk 5 steps with light trunk support from behind	Can walk 5 steps supported by one hand	+2		Can walk 5 steps supported by two hands	+1	
27	Motor function	95% of steps with internal rotation of the right leg	<40% of steps with internal rotation of the right leg	+2	71.0	<40% of steps with internal rotation of the right leg	+2	71.0

28	Motor function	Can climb the stairs with strong support given to both arms and elastic bandages to both knees	Can climb the stairs with light support given to both arms	+1	70.0	Can climb the stairs with strong support given to both arms	0	56.7
	Motor function	Can descend the stairs with strong support given to both arms and elastic bandages to both knees	Can descend the stairs with light support given to one arm	+2		Can descend the stairs with light support given to both arms	+1	
29	Motor function	Can climb the stairs with strong support given to both wrists	Can climb the stairs with support to one wrist and one hand maintained on the rail	0	63.0	Can climb the stairs with support to one wrist and one hand maintained on the rail	0	58.1
	Motor function	Can climb a whole floor of stairs in less than 5 minutes with strong support given to both wrists	Can climb a whole floor of stairs in less than 1 minute with strong support given to both wrists	+1		Can climb a whole floor of stairs in less than 1 minute with strong support given to both wrists	+1	
	Motor function	Can descend the stairs with strong support given to both wrists	Can descend the stairs with one hand maintained on the rail by the caregiver	+1		Can descend the stairs with support to one wrist and one hand maintained on the rail	0	
	Motor function	Can descend a whole floor of stairs in more than 5 minutes with strong support given to both wrists	Can descend a whole floor of stairs in less than 1 minute with strong support given to both wrists	+2		Can descend a whole floor of stairs in less than 1 minute with strong support given to both wrists	+2	
	Motor function	Can participate in the movement of standing up from the floor with strong support given to the trunk	Can participate in the movement of standing up from the floor with strong support given to one wrist	+1		Can participate in the movement of standing up from the floor with strong support given to one wrist	+1	
30	Motor function	Can walk 20 steps with strong trunk support from behind	Can walk 50 steps with strong trunk support from behind	+1	66.8	Can walk 50 steps with strong trunk support from behind	+1	66.8
	Motor function	Can walk 10 steps with light trunk support from behind	Can walk 10 steps supported by two wrists	0		Can walk 10 steps supported by two wrists	0	
	Motor function	Can stand for 20" leaning on a table in front of her	Can stand for >1' leaning on a table in front of her	+2		Can stand for >1' leaning on a table in front of her	+2	

	Motor function	Can sit on the edge of the table for 25" with strong support given to the thighs	Can sit on the edge of the table for >1' with strong support given to the thighs	+2		Can sit on the edge of the table for >1' with strong support given to the thighs	+2	
	Motor function	Can sit on the edge of the table for 15" with light support given to the thighs	Can sit on the edge of the table for 15" with light support given to one thigh	0		Can sit on the edge of the table for 15" with light support given to one thigh	0	
31	Physical fitness	Moderate fear of movement (between 25 and 35 on the fear of movement scale)	Moderate fear of movement (between 15 and 25 on the fear of movement scale)	0	65.0	Moderate fear of movement (between 15 and 25 on the fear of movement scale)	0	30.0
	Motor function	Can stand for 10" independently	Can stand up to 1' independently	+1		Can't stand independently	-2	
	Motor function	Can walk for 5 steps independently	Can walk for more than 20 steps independently	+2		Can walk for 2 steps independently	-2	
32	Motor function	Can climb the stairs with strong support given to both arms	Can climb the stairs with support given to one hand and the other maintained on the rail	+1	46.9	Can climb the stairs with strong support given to the trunk	-2	30.2
	Motor function	Can descend the stairs with strong support given to both arms	Can descend the stairs with strong support given to both wrists	0		Can descend the stairs with strong support given to the trunk	-2	
	Motor function	Can walk up a slope with intermittent support (five steps without the need for support)	Can walk up a slope with intermittent support (five steps without the need for support)	-1		Can walk up a slope without support, with a safety belt	0	
	Motor function	Can walk down a slope with intermittent support (five steps without the need for support)	Can walk down a slope with intermittent support (five steps without the need for support)	-1		Can walk down a slope with intermittent support (five steps without the need for support)	-1	
	Motor function	She can sit in the car with light stimulation to her pelvis and need to be helped to lift both legs in the car	She can sit in the car independently but need to be helped to lift both legs in the car	0		She can sit in the car with light stimulation to her pelvis and need to be helped to lift both legs in the car	-1	
33	Motor function	Can walk >10 steps with light trunk support from behind	Can walk >10 steps supported by two hands	+1	64.9	Can walk >10 steps supported by two hands	+1	59.9

	Motor function	Can walk 3 rounds of the living room without stopping	Can walk 7 rounds of the living room without stopping	+1		Can walk 5 rounds of the living room without stopping	0	
	Range of motion	No deterioration in shoulder flexion (actually -20°both sides)	Improvement of at least 10° (both shoulders)	+1		Improvement of at least 5° (both shoulders)	0	
	Motor function	Can stand for >1' supported by two hands	Can stand for >1' supported by two sleeves	+2		Can stand for >1' supported by two sleeves	+2	
	Motor function	Can stand leaning on the table at her back for less at least 45'' independently	Can stand leaning on the table at her back for at least 2' independently	0		Can stand leaning on the table at her back for at least 2' independently	0	
34	Motor function	Can walk 20 steps with light trunk support from behind	Can walk 100 steps with light trunk support from behind	+2	58.7	Can walk 100 steps with light trunk support from behind	+2	57.0
	Motor function	Can walk 10 steps supported by two wrists	Can walk 10 steps supported by two hands	0		Can walk 10 steps supported by one hand	+1	
	Motor function	Can stand for >1' supported by two wrist	Can stand for >1' held by two sleeves	0		Can stand for >1' supported by two wrist	-1	
	Range of motion	no deterioration (actually -20°)	no deterioration (actually -20°)	-1		no deterioration (actually -20°)	-1	
35	Range of motion	Right shoulder flexion is limited to 90°	Improvement of 10°	+1	63.8	Improvement of 10°	+1	46.5
	Range of motion	Left shoulder flexion is limited to 80°	Improvement of 10°	+1		Improvement of 10°	+1	
	Range of motion	Right shoulder abduction is limited to 94°	Improvement of 10°	+1		Right shoulder abduction is limited to 94°	-1	
	Range of motion	Left shoulder abduction is limited to 86°	Improvement of 10°	+1		Improvement of 5°	0	
	Range of motion	Right hip extension limited to 0°	Improvement of 10°	+1		Improvement of 5°	0	
	Range of motion	Left hip extension limited to 0°	Left hip ext limited to 0°	-1		Left hip ext limited to 0°	-1	
36	Motor function	Can climb the stairs with strong support given to both arms and elastic bandages to both knees	Can climb the stairs with strong support given to both arms	0	55.7	Can climb the stairs with strong support given to both arms	0	57.2

	Motor function	Can descend the stairs with strong support given to both arms and elastic bandages to both knees	Can descend the stairs with strong support given to both arms	0		Can descend the stairs with strong support given to both arms and elastic bandages to both knees	-1	
	Hand functioning	Can eat a piece of food with her hand with complete physical guidance (hand on hand)	Can eat a piece of food with her hand with complete physical guidance (hand on hand)	-1		Can eat a piece of food with her hand with complete physical guidance (hand on hand)	-1	
	Hand functioning	Able to maintain the bottle with both hands while drinking with complete physical guidance (hand-on-hand)	Able to maintain the bottle with both hands while drinking with complete physical guidance (hand-on-hand)	-1		Able to maintain the bottle with both hands while drinking with complete physical guidance (hand-on-hand)	-1	
	Motor function	Can walk independently on the lawn for <1 minute without falling	Can walk independently on the lawn for <5 minutes without falling	+1		Can walk independently on the lawn for <10 minutes without falling	+2	
	Motor function	Can stand up from the chair with support given to one hand and a strong motivational factor (cartoon on tv)	Can stand up from the chair independently with a verbal command	+2		Can stand up from the chair independently with a verbal command	+2	
37	Motor function	Can stand supported by one hand for more than 1'	Can stand independently for more than 1'	+2	64.2	Can stand supported by two sleeves for more than 1'	0	48.2
	Motor function	Can assist the movement of standing up from a high chair (half flexed hip, 45°) when pulled from one wrist	Can stand up independently from a high chair (half flexed hip, 45°) when lightly pulled forward from one hand	0		Can stand up independently from a high chair (half flexed hip, 45°) when lightly pulled forward from one hand	0	
	Motor function	Can sit on a chair from standing with slight support given to pelvis and head	Can sit on a chair from standing with support given to one wrist	+2		Can sit on a chair from standing with support given to two wrists	+1	
	Motor function	Can walk for 5 steps independently	Can walk for 10 steps independently	0		Can walk for 5 steps independently	-1	

38	Motor function	Required up to 1' to stand by herself after she stands up from a chair	She can start walking by herself immediately after standing up from a chair	+2	59.5	She can start walking by herself immediately after standing up from a chair	+2	50.0
	Physical fitness	After 8' of treadmill walking at 1.5km/h with 10° inclination, the pulse is at 130BPM	After 8' of treadmill walking at 1.5km/h with 10° inclination, the pulse is at 110BPM	+1		After 8' of treadmill walking at 1.5km/h with 10° inclination, the pulse is at 110BPM	+1	
	Motor function	She can walk for 15' of treadmill walking at 1.5km/h with 10° inclination	She can walk for 30' of treadmill walking at 1.5km/h with 10° inclination	0		She can walk for 15' of treadmill walking at 1.5km/h with 10° inclination	-1	
	Motor function	Can climb the stairs with strong support given to both arms	Can climb the stairs with strong support given to both wrists	0		Can climb the stairs with strong support given to both wrists	0	
	Motor function	Can descend the stairs with strong unilateral trunk support given from her side	Can descend the stairs with strong unilateral trunk support given from her side	-1		Can descend the stairs with strong unilateral trunk support given from her side	0	
	Motor function	Take 5' to cross the room with an obstacle course	Take less than 1' to cross the room with an obstacle course	+2		Take 5' to cross the room with an obstacle course	-1	
39	Motor function	Can stand leaning on the wall for 40 seconds	Can stand leaning on the wall for 2 minutes	+1	58.0	Can stand leaning on the wall for 2 minutes	+1	67.6
	Motor function	Can sit on an air pillow without support to feet and back for 15 seconds	Can sit on an air pillow without support to feet and back for 30 seconds	0		Can sit on an air pillow without support to feet and back for 30 seconds	0	
	Motor function	Can't turn the head to the right only when passively moved	Can turn the head to the right when called by the caregiver	+1		Can turn the head to the right when called by the caregiver	+1	
	Range of motion	Maintains the head turned to the left 90% of the time	Maintains the head turned to the left 80% of the time	0		Maintains the head turned to the left 50% of the time	+2	
40	Motor function	Can stand for more than one minute with strong support given to the trunk	Can stand for more than one minute with strong support given to both wrists	+1	63.2	Can stand for more than one minute with strong support given to both wrists	+1	55.3
	Motor function	Can stand supported by two wrists for less than 1 minute	Can stand supported by two wrists for 5 minutes	+1		Can stand supported by two wrists for 2 minutes	0	
